# Mismatch repair deficiency and aberrations in the Notch and Hedgehog pathways are of prognostic value in patients with endometrial cancer

**DOI:** 10.1371/journal.pone.0208221

**Published:** 2018-12-06

**Authors:** Genovefa Polychronidou, Vassiliki Kotoula, Kyriaki Manousou, Ioannis Kostopoulos, Georgia Karayannopoulou, Eleni Vrettou, Mattheos Bobos, Georgia Raptou, Ioannis Efstratiou, Dimitrios Dionysopoulos, Kyriakos Chatzopoulos, Sotirios Lakis, Sofia Chrisafi, Dimitrios Tsolakidis, Alexios Papanikolaou, Nikolaos Dombros, George Fountzilas

**Affiliations:** 1 Department of Medical Oncology, Papageorgiou Hospital, Aristotle University of Thessaloniki, School of Health Sciences, Faculty of Medicine, Thessaloniki, Greece; 2 Department of Pathology, Aristotle University of Thessaloniki, School of Health Sciences, Faculty of Medicine, Thessaloniki, Greece; 3 Laboratory of Molecular Oncology, Hellenic Foundation for Cancer Research/Aristotle University of Thessaloniki, Thessaloniki, Greece; 4 Section of Biostatistics, Hellenic Cooperative Oncology Group, Data Office, Athens, Greece; 5 Department of Pathology, Papageorgiou Hospital, Thessaloniki, Greece; 6 First Department of Obstetrics and Gynaecology, Papageorgiou General Hospital, Aristotle University of Thessaloniki, Thessaloniki; 7 Aristotle University of Thessaloniki, Thessaloniki, Greece; University of South Alabama Mitchell Cancer Institute, UNITED STATES

## Abstract

The aim of this study was to investigate the prognostic value of the Hedgehog (Gli, Patched-1, Shh, Smo) and Notch (Jag1, Notch2, Notch3) pathway members, in comparison to a panel of proteins (ER, PgR, HER2/neu, Ki67, p53, p16, PTEN and MMR) previously suggested to be involved in the pathogenesis of endometrial cancer, in association with clinical outcome and standard clinicopathological characteristics. A total of 204 patients with histological diagnosis of endometrial cancer treated from 2004 to 2013 were included. The evaluation of protein expression was assessed by immunohistochemistry. Univariate analysis showed that higher Ki67 labeling, expression of PTEN, p16, Notch2 and Notch3 proteins, as well as MMR proficiency were associated with increased relapse and mortality rate. Additionally, Patched-1 protein expression was associated with worse DFS, while p53 overexpression was associated with worse OS. In multivariate analyses, patients with MMR proficient tumors had more than double risk for death than patients with MMR deficient (MMRd) tumors (adjusted HR = 2.19, 95% CI 1.05–4.58, p = 0.036). Jag1 positivity conferred reduced mortality risk (HR = 0.48, 95% CI 0.23–0.97, p = 0.042). However, as shown by hierarchical clustering, patients fared better when their tumors expressed high Jag1 protein in the absence of Notch2 and Notch3, while they fared worse when all three proteins were highly expressed. Patched-1 positivity conferred higher risk for relapse (HR = 2.04, 95% CI 1.05–3.96, p = 0.036).

Aberrant expression of key components of the Notch and Hedgehog signaling pathways, as well as MMRd may serve as independent prognostic factors for recurrence and survival in patients with endometrial cancer.

## Introduction

Endometrial carcinoma is the most common malignancy of the female reproductive tract with a substantial increase in incidence and mortality rates in the developed countries [1]. Historically, endometrial cancer has been categorized into two subgroups, described by Bokhman approximately 30 years ago [2]. Type I carcinomas are typically characterized by low-grade endometrioid histology, hormone receptor positivity, early stage at diagnosis and favorable prognosis and account for the majority of the cases (70–80%), while type II carcinomas are associated with non-endometrioid histology, advanced stage at the time of diagnosis, higher risk for metastases and poor prognosis. The most frequent molecular alterations in type I endometrial carcinomas include PTEN loss of function, PIK3CA mutations, microsatellite instability (MSI) and KRAS mutations. Respectively, type II endometrial carcinomas demonstrate p53 mutations, overexpression of HER2/neu, p16 loss of function and aneuploidy [3].

However, this system of taxonomy exhibits marked heterogeneity and has limited clinical utility in risk determination, as tumors with common phenotypes can have different gene expression and clinical outcome. Therefore, an effort has been made to incorporate molecular features into classification and risk stratification of endometrial cancers, with the ultimate goal to better assess the biological aggressiveness of the disease and improve guidance on management decisions [4]. The Cancer Genome Atlas (TCGA) project provided the most comprehensive molecular study of endometrial cancer, which classified 232 endometrioid and serous endometrial cancers into four groups, POLE ultramutated, MSI hypermutated, copy-number low and copy-number high, associated with differential progression-free survival [5]. Nevertheless, due to the high cost and low reproducibility, the method is currently not applied in routine clinical practice.

The Notch signaling pathway is an evolutionally conserved signaling system that regulates differentiation in embryonic and postnatal tissues and determines cell fate and proliferation [6]. Notch2 and Notch3 are two of the four mammalian Notch receptors (Notch 1–4), while Jagged 1 (Jag1) is one of the five ligands (Jag1-2, Delta-like 1, DLL3 and DLL4) [7]. Aberrant activation of the Notch pathway has been documented in a variety of malignancies, where it might have pro-tumorigenic but also tumor suppressive functions [8]. Experimental and preliminary clinical targeting of the Notch pathway [7], particularly of Jag1 [9] [10], has been shown to be promising in multiple cancer types. In endometrial cancer however, aberrations of the Notch pathway may have a tumor suppressive effect [11].

Hedgehog is another fundamental developmental pathway that has been shown to play a crucial role in cell fate regulation, differentiation and proliferation, as well as stem cell maintenance [12]. Constitutive activation of the Hedgehog pathway has been implicated in tumorigenesis and may be involved in the early events of endometrial carcinogenesis [13]. The Hedgehog receptor Patched-1 is a negative regulator of the pathway and acts by blocking the Smoothened activity [14], whereas Sonic Hedgehog (Shh) is one of the three ligands (Indian and Desert) binding to the Patched-1 receptor and resulting in the activation of the Hedgehog signaling cascade [12]. In the few relevant studies, Hedgehog pathway proteins are overexpressed in endometrial cancer [15] [16] and appear to be targeted by the antifungal agent itraconazole [16].

Taking into account the need for emerging reliable prognostic and/or predictive markers in treating endometrial cancer, current research focuses on the use of more pragmatic approaches. In line with this, and also given the limited information on the role that Hedgehog and Notch pathway aberrations may play in endometrial cancer, we performed a protein expression analysis of the Hedgehog and Notch pathway members in 204 endometrial carcinomas, by using immunohistochemistry (IHC) on tissue microarrays (TMAs). The aim of this study was to determine the potential prognostic value of Hedgehog and Notch pathway aberrations in endometrial cancer and to examine their association with clinical outcome and other clinicopathological characteristics, including a panel of protein markers (ER, PgR, HER2, Ki67, p53, p16, PTEN and mismatch repair [MMR] proteins) that had previously been implicated in the pathogenesis of endometrial cancer or had demonstrated significance as prognostic markers [17].

## Patients and methods

### Patient cohort

The present study included endometrial carcinomas obtained from 204 women surgically treated at Papageorgiou Hospital or referred to the Department of Oncology, Aristotle University of Thessaloniki, School of Medicine, from 2004 to 2013. Formalin-fixed paraffin-embedded (FFPE) tissue blocks were retrieved from the Hellenic Cooperative Oncology Group (HeCOG) tissue repository. All tissues were primary tumors obtained before any treatment. We retrospectively recorded the clinicopathological characteristics, treatment information and follow-up data. The classification of endometrial tumors into the two subtypes of endometrial cancer (type I and type II) was based on histomorphologic criteria determined by the histology report. Tumors of endometrioid histology were characterized as type I, while tumors of non-endometrioid histology (serous, clear cell, mixed and undifferentiated) were characterized as type II. All patients included in the study had signed informed consent for the use of their biological material for future research. The study was performed according to the principles of the Declaration of Helsinki and was approved by the Ethics Committee of the Aristotle University School of Medicine.

### Tissue processing

Hematoxylin-eosin stains from each FFPE block were evaluated by a pathologist in order to verify all tumor characteristics and choose the tumor region to be used for tissue microarray construction. TMAs were constructed with a manual arrayer (Model I, Beecher Instruments, Sun Prairie, WI, USA), with 2X1.5 mm cores per tumor along with various neoplastic and reactive tissues for orientation.

### IHC methods

Serial 3 micron thick sections from the TMA blocks were cut and mounted on adhesive microscope slides. Immunohistochemical staining for the various markers has been performed using the Bond MaxTM (Leica Microsystems, Wezlar, Germany) and the Bond Polymer Refine Detection kit (DS9800, Leica Biosystems). Staining and evaluation methods for all antibodies are shown in detail in S1 Table.

### IHC evaluation and scoring system

Staining intensity for Notch2, Notch3 and Jag1 was scored into four grades (0, 1+, 2+ and 3+), while the percentage of positive cells was also scored into four categories (0 for 0%, 1 for 1–33%, 2 for >33–66% and 3 for >66–100%). The product of intensity and percentage categories (range 0–9) was finally classified as negative (0–4) and positive (5–9) [18]. Similarly, for Gli, Patched-1, Shh and Smo, intensity was scored into four grades (0, 1+, 2+ and 3+), while the percentage of positive cells was scored into five categories (0 for <5%, 1 for 5–25%, 2 for >25–50%, 3 for >50–75% and 4 for >75%). The product of the intensity and percentage categories (range 0–12) was used as the final score and classified as negative (0–2) and positive (3–12) [19].

Positivity for ER and PgR values was considered for ≥1% positive cells at any intensity [20, 21], for p53 for values ≥75% [22], for p16 for ≥10% [23] and for HER2 for >10% [24] at any intensity. PTEN evaluation was based on tumor and internal positive control staining and was interpreted as: 1. Positive, when strong positive staining in the entire tumor or vast majority of the tumor was present; 2. Negative, when no staining was observed in the entire or vast majority of the tumor, while the internal control cells were strongly positive; 3. Heterogeneous, when tumors had both above staining patterns (positive and negative) within the sampled core areas. A histologic score was calculated for the heterogeneous cases according to Garg K. et al [25] and finally the tumors were defined as PTEN loss/PTEN no loss. Tumors with ≥15% positive cells for Ki67 were considered as high expression [26]. MMR evaluation was based on the detection of four MMR proteins (MLH1, MSH2, MSH6 and PMS2), which were considered positive if ≥10% positive nuclei with mild to strong intensity were encountered. Tumors with a negative result in one of the four proteins were characterized as MMR deficient (MMRd) [27]. Study markers were also used as continuous measurements of percent positivity, where such data were available. For the p53, HER2, PTEN and MMR markers continuous measurements were not available.

**Fig 1:**
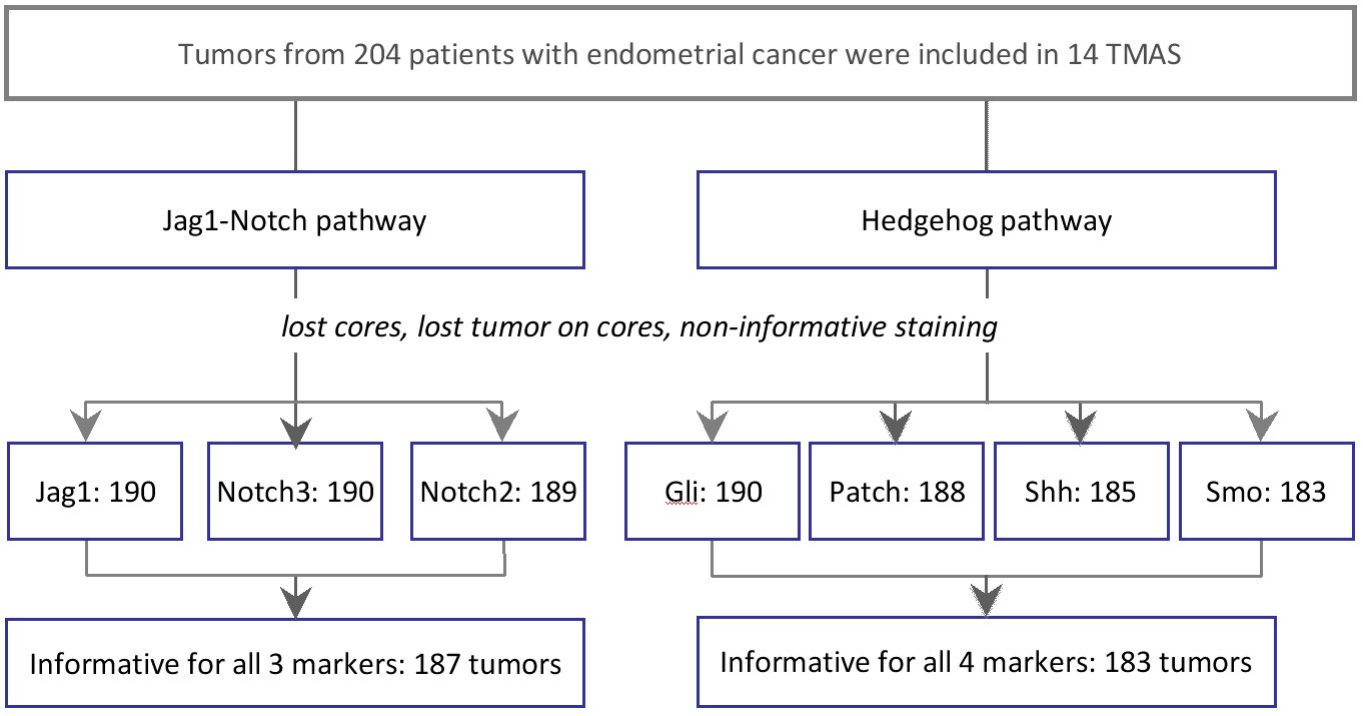
Study outline and informative tumors for each marker and pathway. TMA: tissue microarray.

**Fig 2:**
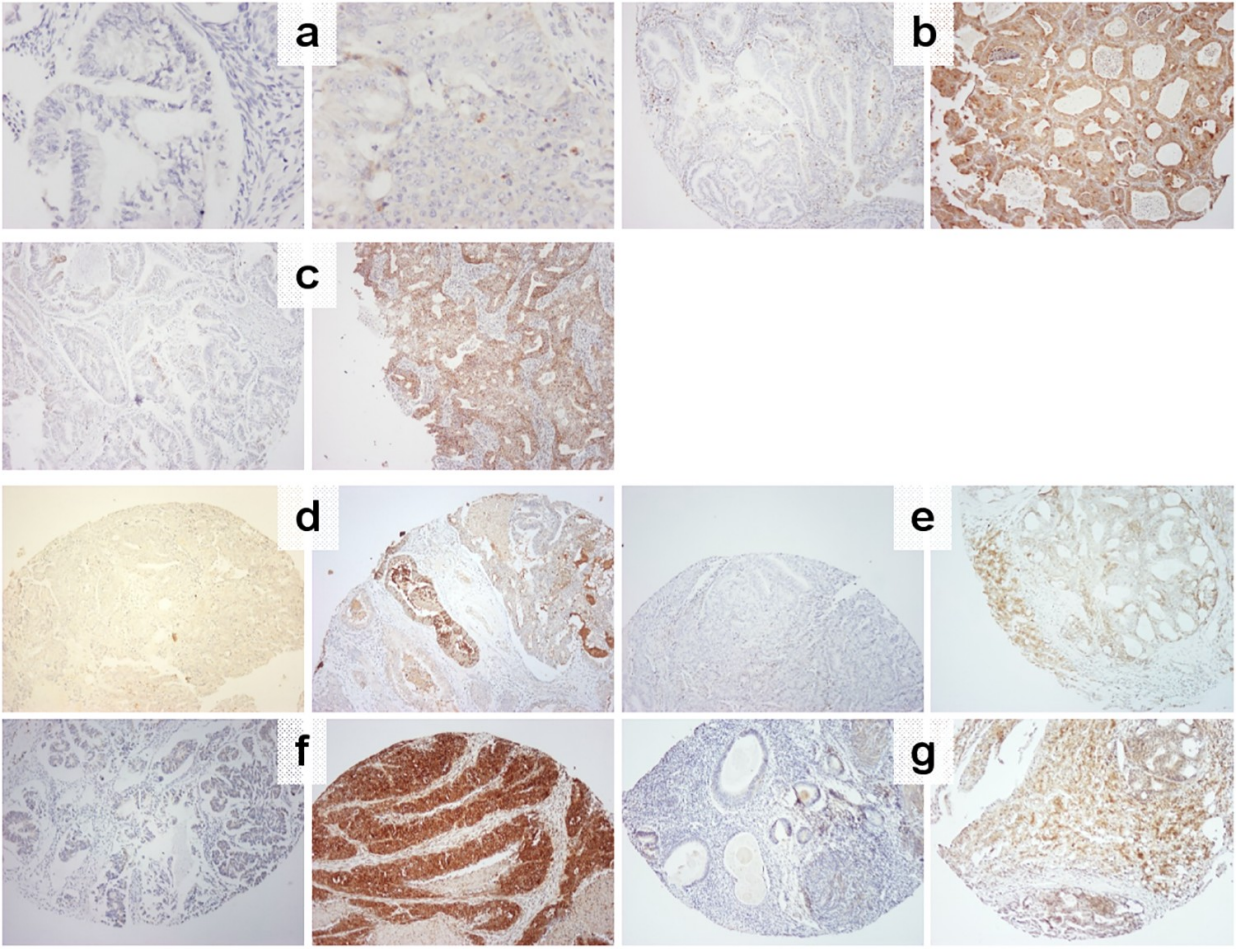
Characteristic examples of negative and positive protein markers with IHC. For all panels, negative is on the left, positive on the right. (a) Notch2, x400; (b) Notch3, x100; (c) Jag1, X100; (d) Gli, X100; (e) Patched-1, X100; (f) Shh, X100; (g) Smo, X100.

The study outline and the number of informative tumors that were processed for analysis are shown in Fig 1. IHC examples of the Notch and Hedgehog pathway markers tested in this study are shown in Fig 2. Examples for all other markers are shown in S1 Fig.

### Statistical methods

A total of 204 patients with endometrial cancer were included in the present study. Continuous variables are presented as medians (range) and categorical variables as frequencies (%). The chi-square test was used for group comparisons of categorical data.

Overall survival (OS) was defined as the time (in months) from the date of diagnosis with endometrial cancer to the date of the patient’s death or last contact, while disease-free survival (DFS) as the time (in months) from the date of diagnosis to documented first relapse, death without prior documented relapse or last contact, whichever occurred first. Surviving patients were censored at the date of last contact. Women who died without prior relapse were treated as events, i.e., as having relapsed at the date of their death. OS and DFS at 5 years were the primary endpoints. Survival curves were estimated using the Kaplan-Meier method and compared across groups with the log-rank test. The associations between the factors examined and relapse/mortality rates were evaluated with hazard ratios estimated with the Cox proportional hazards model.

The following parameters were studied in relation to 5-year DFS and OS: (1) clinicopathological characteristics, such as age, endometrial cancer type, grade, depth of invasion, stage, adjuvant chemotherapy and radiotherapy and (2) IHC markers, such as ER, PgR, HER2, p53, p16, Ki67, PTEN, Jag1, Notch2, Notch3, Gli, Patched-1, Shh, Smo and MMR status.

In multivariate analyses, we estimated the effect (hazard ratio, HR) of each IHC marker adjusted for the effect of the clinicopathological parameters that were statistically significant in the univariate analysis.

In addition, unsupervised hierarchical clustering, using the Ward’s minimum variance method, was employed in order to identify distinct groups with common biological characteristics for the following subgroups of IHC markers with continuous measurements: 1) Jag1, Notch2 and Notch3 (i.e. Jag1-Notch pathway) and 2) Gli, Patched-1, and Smo (i.e. Hedgehog pathway). Shh was not included in the clustering, since almost all tumors in our sample overexpressed this marker. Standardized values for each marker were used for estimating the cluster distances. The selection of the optimal number of clusters was based on pseudo F-statistics, which describe the ratio of the between-cluster variance and within cluster variance.

All analyses were performed in the entire cohort. The statistical analyses were performed using the SAS software (SAS for Windows, version 9.3, SAS Institute Inc., Cary, NC). Statistical significance was set at 2-sided p = 0.05. Results of this study were presented according to reporting recommendations for tumor marker prognostic studies [28].

## Results

Clinicopathological characteristics and IHC markers of the entire cohort are presented in Tables 1 and 2, whereas characteristics of type I and type II tumors are presented in S2 Table. 72.6% of the patients presented with endometrial cancer of stage I-II, while type I tumors accounted for 80.4% of the cases. The majority of tumors had positive ER/PgR status, negative HER2 status, and no p53 protein overexpression. Additionally, high Ki67 and PTEN loss were observed in the majority of tumors. Regarding the Jag1-Notch pathway, more than 70% of the tumors were negative for all three proteins with the cut-offs used for IHC. Regarding the Hedgehog pathway, the majority of tumors did not express the Gli, Patched-1 and Smo proteins, however almost all of the tumors (97.3%) were Shh-positive.

**Table 1:** Selected patient and tumor characteristics, N = 204.

Parameter	N (%)
**Age**	
Median (range)	63.9 (29–87)
**Stage**	
Early	145 (71.1)
Advanced/Relapsed	59 (28.9)
**Stage**	
I	135 (66.2)
II	13 (6.4)
III	36 (17.6)
IV	14 (6.8)
Not reported	6 (3.0)
**Type**	
I	164 (80.4)
II	37 (18.1)
Not reported	3 (1.5)
**Grade**	
1	53 (26.0)
2	89 (43.6)
3	58 (28.4)
Not reported	4 (2.0)
**Depth of invasion**	
<50%	97 (47.6)
≥50%	99 (48.6)
Not reported	8 (4.0)
Adjuvant chemotherapy*	
Yes	24 (11.8)
No	178 (86.2)
Not reported	2 (0.8)
**Adjuvant radiotherapy**	
Yes	121 (59.4)
No	82 (40.2)
Not reported	1 (0.4)

*paclitaxel/carboplatin

**Table 2:** Immunohistochemical results for all markers.

Parameter	N (%)	Parameter	N (%)
**ER status**		**Jag1 status**	
*Informative*	*191 (93*.*6)*	*Informative*	*189 (92*.*6)*
Negative	64 (33.5)	Negative (0–4)	134 (70.9)
Positive	127 (66.5)	Positive(5–9)	55 (29.1)
**PgR status**		**Notch2 status**	
*Informative*	*189 (92*.*6)*	*Informative*	*190 (93*.*0)*
Negative	53 (28.0)	Negative (0–4)	153 (80.5)
Positive	136 (72.0)	Positive(5–9)	37 (19.5)
**HER2 status**		**Notch3 status**	
*Informative*	*189 (92*.*6)*	*Informative*	*190 (93*.*0)*
Negative	124 (65.6)	Negative (0–4)	166 (87.4)
Positive	65 (34.4)	Positive(5–9)	24 (12.6)
**p53 status**		**Gli (cut-off at 3)**	
*Informative*	*191 (93*.*6)*	*Informative*	*190 (93*.*0)*
No overexpression	143 (74.9)	Negative	131 (69.0)
Overexpression	48 (25.1)	Positive	59 (31.0)
**p16**		**Patched-1 (cut-off at 3)**	
*Informative*	*183 (89*.*7)*	*Informative*	*188 (92*.*0)*
Negative	97 (53.0)	Negative (0–2)	126 (67.0)
Positive	86 (47.0)	Positive (3–12)	62 (33.0)
**Ki67 status**		**Shh (cut-off at 3)**	
*Informative*	*187 (91*.*7)*	*Informative*	*185 (90*.*7)*
Low	70 (37.4)	Negative (0–2)	5 (2.7)
High	117 (62.6)	Positive (3–12)	180 (97.3)
**PTEN status**		**Smo (cut-off at 3)**	
*Informative*	*188 (92*.*2)*	*Informative*	*183 (89*.*7)*
Loss	117 (62.2)	Negative (0–2)	112 (61.2)
No loss	71 (37.8)	Positive (3–12)	71 (38.8)
**MMR status**			
*Informative*	*178 (87*.*3)*		
Deficiency	81 (45.5)		
Proficiency	97 (54.5)		

The associations between IHC markers and basic clinicopathological parameters are presented in S3 Table. In comparison to type II (N = 37), type I tumors (N = 164) more frequently: were PgR-positive (chi-square, p<0.001); expressed low Ki67 (p = 0.014) and p53 (p<0.001); did not express p16 (p<0.001), Notch2 (p<0.001) and Notch3 (p = 0.004); demonstrated PTEN loss (p<0.001); and, were MMRd (p = 0.003). In comparison to advanced stage (III-IV, N = 50), tumors of lower stage (I-II, N = 148) more frequently: were PgR-positive (p = 0.001); expressed low Ki67 (p = 0.006) and low p53 (p = 0.001); did not express p16 (p = 0.001), Notch2 (p<0.001) and Notch 3 (p<0.001); and, were MMRd (p = 0.031). Finally, as compared to grade 1 (N = 53) or 2 (N = 89), grade 3 tumors (N = 58) more frequently: were PgR-negative (p = 0.009); expressed high Ki67 (p<0.001); overexpressed p53 (p<0.001); expressed p16 (p<0.001), PTEN (p<0.001), Notch2 (p<0.001), Notch3 (p<0.001) and Patched-1 (p = 0.045); and, were MMR proficient (p = 0.007).

The associations among the IHC markers are presented in S4 Table. Notch2-positive tumors also expressed Notch3 (p<0.001); Notch2- and Notch3-positive tumors were more frequently Gli-negative (p = 0.030 and 0.035, respectively); and, Jag1-positive tumors were more frequently Patched-1-negative (p = 0.038). Using the continuous measurements of the IHC markers, Jag1 was positively correlated with Patched-1 (Spearman’s rho = 0.27) and Shh (rho = 0.25); Notch2 was positively correlated with Patched-1 (rho = 0.28) and Shh (rho = 0.24), while it was negatively correlated with Gli (rho = -0.28). Finally, Notch3 was positively correlated with Patched-1 (rho = 0.36), Shh (rho = 0.24) and Smo (rho = 0.22). However, in all these correlations, the coefficient (rho) was very low to infer any biological significance.

### Effect of single study parameters on outcome

In S5 Table, the follow-up time, OS, DFS and relapse/mortality rates during follow-up are presented. During a median follow-up of 72 months, 59 deaths (28.9%) and 45 relapses (22.1%) occurred. Within the first 5 years, 52 (25.5%) deaths and 42 (20.6%) relapses occurred.

Results from univariate Cox regression analyses in the entire cohort are presented in S6 Table. Beyond classic unfavorable prognostic factors in endometrial cancer, such as tumor type II, higher grade and more advanced stage, higher Ki67 labeling, expression of the PTEN, p16, Notch2 and Notch3 proteins, as well as MMR proficiency were associated with increased relapse and mortality risk. Additionally, Patched-1 protein expression was associated with worse DFS and p53 overexpression with worse OS.

Results from multivariate analyses including the aforementioned clinicopathological parameters and each of the IHC markers are presented in S7 Table. Grade has retained independent prognostic significance for both DFS and OS, whereas stage only for OS. This is not surprising, as grade of tumor differentiation is a well-established marker of prognosis and has been associated with depth of myometrial invasion and lymph node involvement [29]. The HRs associated with MMR proficiency, Jag1, Notch2 and Patched-1 positivity adjusted for the rest of the clinicopathological variables were of similar magnitude as the unadjusted HRs, both for DFS and OS. Patients with MMR proficient tumors had more than twice the risk for relapse/death than patients with MMR deficient tumors (DFS: HR = 2.26, 95% CI 0.99–5.18, p = 0.054 and OS: HR = 2.19, 95% CI 1.05–4.58, p = 0.036). Jag1 positivity, as a single marker, demonstrated a trend for reduced risk for relapse (adjusted HR = 0.50, 95% CI 0.23–1.09, p = 0.081) and was independently associated with reduced risk for death (HR = 0.48, 95% CI 0.23–0.97, p = 0.042). In comparison to Jag1, Notch2 exhibited a trend in the opposite direction with respect to relapse (HR = 1.93, 95% CI 0.90–4.13; p = 0.093).

Regarding the Hedgehog pathway, Patched-1 positivity independently conferred increased risk for relapse (HR = 2.04, 95% CI 1.05–3.96, p = 0.032).

### Cluster analysis

**Fig 3:**
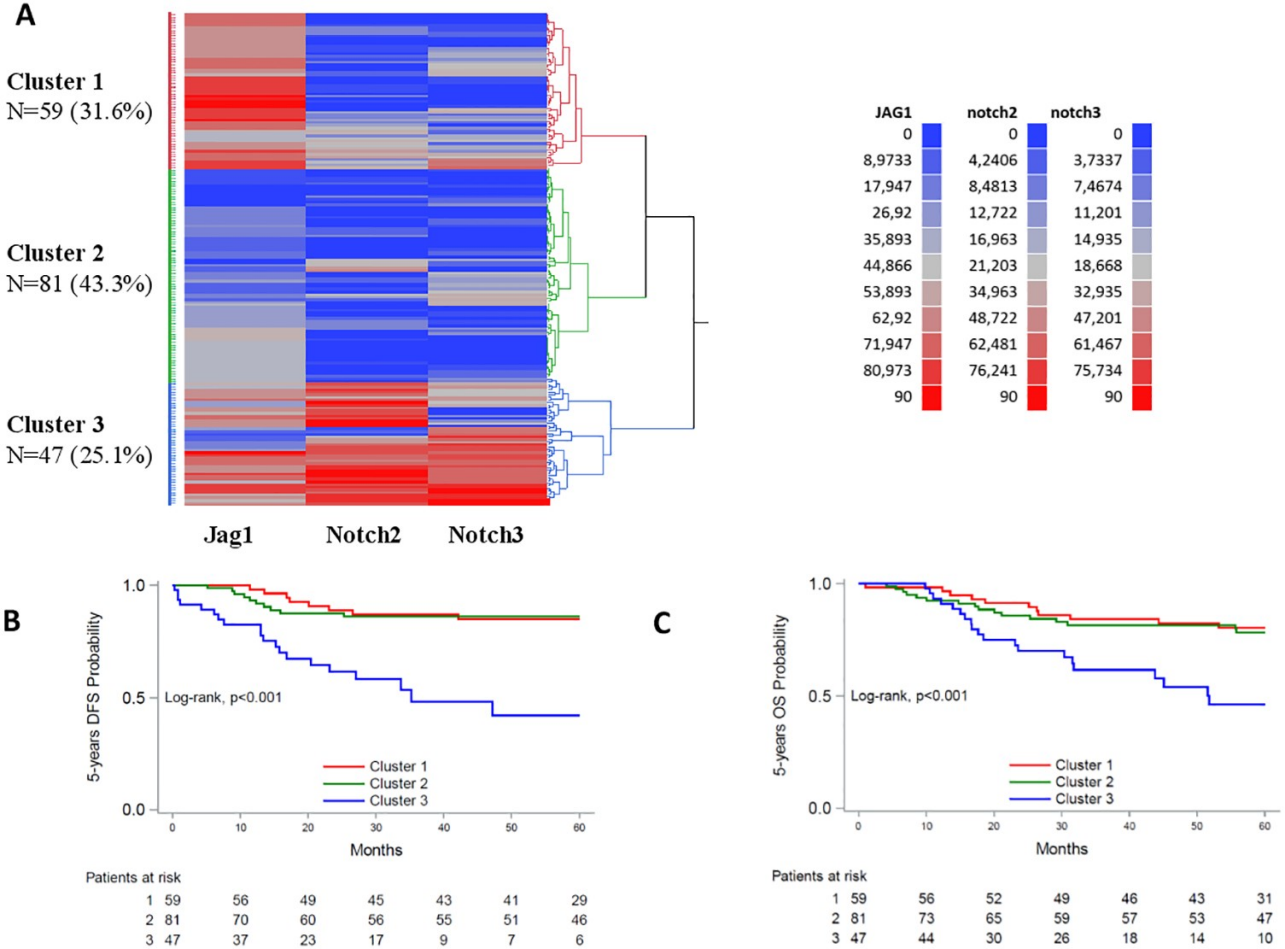
Evaluation of Jag1-Notch pathway activation patterns. A: Hierarchical clustering of continuous IHC measurements, N = 187. B, C: Kaplan-Meier curves according to cluster membership. B: 5-year DFS; C: 5-year OS.

**Fig 4:**
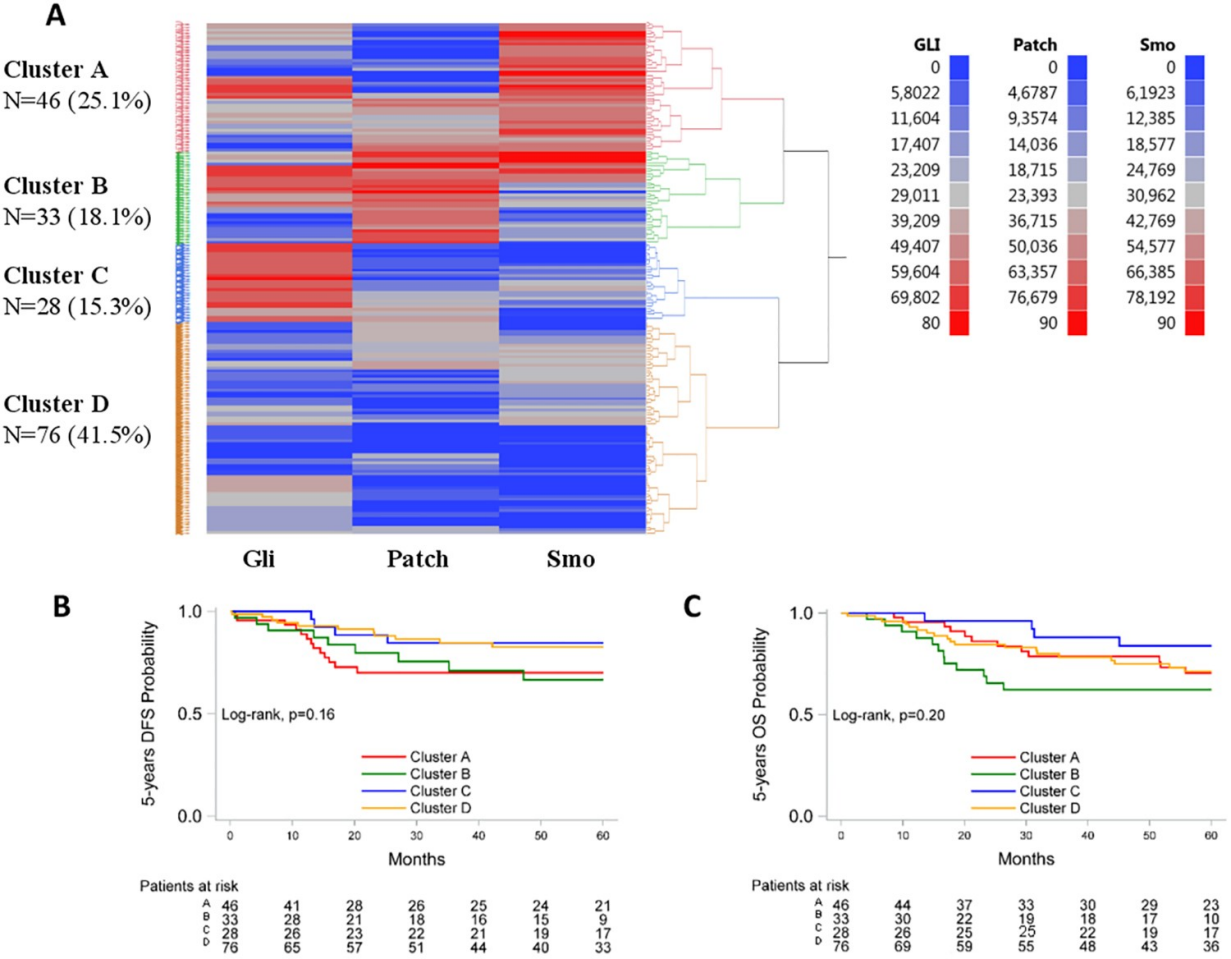
Evaluation of the Hedgehog pathway activation patterns. A: Hierarchical clustering of continuous IHC measurements for the assessment of the Hedgehog pathway activity patterns, N = 183. B, C: Kaplan-Meier curves according to cluster membership. B: 5-year DFS; C: 5-year OS.

We hypothesized that the expression patterns of pathway protein members might be more informative, reflecting the status of the respective pathway, than the expression of each protein individually. In order to explore this hypothesis, we next applied hierarchical clustering for the markers with continuous measurements. Three clusters were distinguished for the Notch pathway (Jag1/Notch2/Notch3) (Fig 3A). In cluster 1, Jag1 was relatively highly expressed compared to Notch2/Notch3 that were low to undetectable; in cluster 2, all three markers were low to undetectable; in cluster 3, all three markers were highly expressed. Four clusters were distinguished for the Hedgehog pathway (Gli/Patched-1/Smo) (Fig 4A). In cluster A, tumors were characterized by relatively high Smo expression; in cluster B, by relatively high Patched-1 expression; in cluster C, Gli expression dominated over Patched-1 and Smo; in cluster D, the expression of all three markers was low to undetectable. The median values of the markers in the Notch and Hedgehog clusters are presented in S2 Fig.

The associations between the identified clusters and the IHC markers, as well as the clinicopathological parameters are presented in S8 Table. As compared to tumors in clusters 1 and 2, tumors overexpressing all three Jag1/Notch2/Notch3 (cluster 3) were more frequently PgR- and Gli-negative (overall chi-square p<0.001 and p = 0.012, respectively); expressed PTEN (p = 0.003); expressed high Ki67 (p<0.001); overexpressed p53 (p = 0.002); were p16- (p<0.001) and Patched-1-positive (p = 0.016); and, were MMR proficient (p = 0.007). Additionally, tumors in cluster 3 were less frequently of type I and more frequently of higher stage (III-IV) and of histological grade 3 (all p-values <0.001).

Regarding the four Gli/Patched-1/Smo clusters, tumors in cluster D (expression of all three markers low to undetectable), as compared to tumors in the other three clusters, were more frequently ER-negative (p = 0.017), while tumors in cluster B (Patched-1 overexpressed) more frequently expressed p16 (p = 0.038) and PTEN (p = 0.019). Finally, tumors in cluster C (Gli overexpressed) had more frequently low Ki67 labeling (p<0.001) and did not exhibit p53 (p = 0.001) overexpression, while all tumors in this cluster were of type I (p = 0.038).

When examining the three Jag1/Notch2/Notch3 clusters with respect to outcome, patients with tumors overexpressing all three markers (cluster 3) had significantly increased risk for relapse (cluster 1 vs. 3, HR = 0.23, 95% CI 0.10–0.52, p<0.001; cluster 2 vs. 3, HR = 0.20, 95% CI 0.10–0.45, p<0.001) and death (cluster 1 vs. 3, HR = 0.34, 95% CI 0.16–0.72, p = 0.005; cluster 2 vs. 3, HR = 0.32, 95% CI 0.16–0.65, p = 0.002) (Fig 3B and 3C). Of note, the favorable effect of dominant Jag1 expression (cluster 1) disappeared in the presence of simultaneous relatively high Notch2 and Notch 3 expression (cluster 3). When adjusted for the effect of tumor type, grade, stage and depth of invasion, high Jag1/Notch2/Notch3 (cluster 3) expression remained an unfavorable prognostic factor for DFS when compared to high Jag1 without Notch2/Notch3 (cluster 1) or when all 3 proteins were low (cluster 2), although these results were of marginal statistical significance (cluster 1 vs. 3, adjusted HR = 0.40, 95% CI 0.15–1.02, p = 0.055; cluster 2 vs. 3, adjusted HR = 0.40, 95% CI 0.15–1.07, p = 0.067). No significant differences among the clusters with respect to OS were observed.

When examining the four Gli/Patched-1/Smo clusters with respect to outcome, no significant differences in relapse rates were observed (Fig 4B). With respect to OS, tumors overexpressing Gli (cluster C) were associated with reduced mortality rates compared to tumors overexpressing Patched-1 (cluster B) (HR = 0.30, 95% CI 0.10–0.96, p = 0.042) (Fig 4C). However, when adjusting for clinicopathological variables no significant difference between the two clusters was observed.

## Discussion

In the present series of patients with endometrial cancer of all types and stages we found that Notch2 and Jag1 protein expression has opposite prognostic impact. In a recent study, Devor et al. reported that recurrence in patients with endometrial cancer of endometrioid histology (type I) is in part attributable to an upregulation of Notch2, modulated by a down-regulation of miR-181c [30]. Out findings extend the unfavorable prognostic impact of Notch2 protein expression in both types of endometrial cancer, which may be related to the herein observed associations between Notch2 and Notch3 with aggravating endometrial cancer characteristics, i.e., advanced disease stage, type II carcinomas and poor histological differentiation. Although expression of Notch3 has previously been associated with an aggressive tumor phenotype, senescence induction, and self-renewal of tumor-propagating cells in cancer [6, 8], in the present study Notch3 protein expression did not yield statistically significant results in terms of outcome, possibly due to the small number of Notch3-positive patients in our cohort.

The novel finding from this study, with potential clinical research relevance regarding the Notch pathway, is with respect to Jag1 protein expression. Jag1 is reported as tumor promoting in many cancer types [31] and on this basis, it is considered to be a target for anti-cancer agents [10] [32]. Our data on Jag1 expression, as an individual binary marker, reflected a favorable prognostic impact of this protein, which may be considered compatible with the proposed tumor-suppressing role of Notch pathway activation in endometrial cancer [33] [11] and the dual role of Notch activation in cancer [34] [6] [8]. However, as we have shown, this effect of Jag1 may depend on the presence of Notch2/3: in our study, high expression of the Jag1 ligand was favorable in the absence of Notch2/3, but unfavorable in the presence of these receptors. In fact, the outcome of patients with Jag1 expression without Notch2/3 was very similar to that of patients with low or undetectable expression of all three markers, while in both these instances patients fared better compared to those with tumors expressing all three markers at high levels. It is reasonable to infer that the pathway is likely activated in the latter cases. On this ground, activation of the Notch pathway was associated to unfavorable prognosis in our patients with endometrial cancer. Overall, these data indicate that Notch pathway proteins should not be assessed individually, but rather examined in a context-dependent and cell type-specific manner, as previously suggested [34]. Clearly, our Jag1 and Notch2/3 findings should be regarded as hypothesis generating and indisputably need further investigation, especially in view of the development of Notch pathway inhibitors, where candidate patients will need to be selected [35] [32].

In the present study Shh was overexpressed in almost all tumors of the cohort, while Patched-1, Smo and Gli were expressed in approximately one-third of the cases in different combinations, as shown by our clustering approach. A previous study regarding expression patterns of members of the Hedgehog pathway in breast carcinoma tissues revealed that nuclear expression of Gli was associated with ER-positive cases, implying that the Hedgehog pathway may be involved in the hormone-induced carcinogenesis of this tumor [36]. Likewise, we have demonstrated that all the major Hedgehog pathway components examined were significantly associated with ER-positive status. These results suggest that the Hedgehog pathway may be linked to endometrial carcinogenesis through hormonal stimulation of the endometrium.

Patched has been suggested to have a tumor-suppressing function, and loss of function mutations or down-regulation of the gene have been implicated in the tumorigenesis of several malignancies [37]. Overexpression of Patched-1 has been reported in uterine and endometrial carcinoma [15, 19], while Gli1 siRNA has been shown to suppress the expression of Patched-1 and induce apoptosis in Huh7 cells [38]. Further, low Patched-1 expression has been linked to a favorable outcome in some cancer types, i.e. colorectal [39] and esophageal squamous cell carcinomas [40]. In line with the findings in gastrointestinal cancer, we have demonstrated that Patched-1 protein expression significantly correlates with worse DFS and is more frequently positive in poorly differentiated tumors. This result could be linked with the fact that ectopic expression of Patched-1 might alter its normal inhibitory function on Smo, resulting in the aberrant activation of the Hedgehog pathway, as well as the downstream transcription factor Gli [41]. The pro-oncogenic role of Patched-1 and/or its association with the administered treatment merits functional validation and confirmation in larger series of endometrial cancer.

Development of therapeutics regarding the Shh signaling pathway has primarily focused on targeting Smo and Gli. Preclinical experiments have already shown efficacy of single-agent Smo inhibitors in some malignancies, while two of these active agents have received FDA approval for treating advanced or metastatic basal cell carcinoma [42]. In addition, Shh inhibitors have been shown to successfully inhibit the Shh pathway, although they have not reached the clinic yet. The above findings provide a viable rationale for preclinical evaluation of these molecules in endometrial cancer [43].

Mismatch repair deficiency (MMRd) has been reported in 20–30% of endometrial cancers. Yet, in contrast to colorectal cancer, there are limited data across the literature regarding the prognostic and predictive impact of MMRd in endometrial cancer. Several reports have suggested improved outcomes for patients with MMRd endometrial tumors [44–48]. We show that MMRd was associated with significant better survival but not with decreased risk for relapse, which is fully in line with the majority of these reports [44, 46, 47]. Contradictory results indicating decreased survival or no difference in prognosis or risk for relapse in association with MMRd [49–54] have also been published, including a meta-analysis involving 23 studies [53]. Such inconclusive results may be due to the variety of methods used for the assessment of MMRd in tumor tissues and the different clinical settings examined, particularly with respect to disease stage and endometrial tumor type. From the biological point of view, MMRd tumors are hypermutated [5] [55] and produce high loads of neoantigens triggering the host immune response [56] [55]. These data provide a rationale for a better outcome in MMRd endometrial cancers. Whether this feature is stage and tumor type-dependent, it needs to be tested in large studies for increased statistical power, as well as methodologically homogenous assessment of MMR status. Based on the recent development that MMRd status should be assessed across cancers as predictive of response to immunotherapy [57] [58], which led to the first tumor-agnostic approval for such treatments by the FDA in August 2017, the clinical relevance of MMRd in endometrial cancer is worth studying for further refinement.

From the methodological point of view, the present study is one of the few published in the literature to apply hierarchical clustering for protein expression in endometrial cancer [17]. We identified previously not reported distinct groups with common biological characteristics and potential prognostic relevance for the Jag1-Notch and Hedgehog pathways, suggesting that the use of multiple markers is superior to that of single markers. Since clinically relevant and easily reproducible sub-classification of endometrial tumors is warranted, further validation of these findings with carefully designed and well-conducted prospective trials incorporating evaluation of the appropriate biomarkers is prompted.

One limitation of our study is the retrospective collection of tissue samples, although central histology review of each case was performed. Moreover, there are IHC technique limitations, as standardized protocols for the use of the assessed monoclonal antibodies in endometrial cancer do not exist. Nevertheless, this is to our knowledge one of the largest studies that evaluates a wide range of protein markers in endometrial cancer.

In conclusion, we have found that aberrant expression of key components of the Notch and Hedgehog signaling pathways, as well as MMRd may serve as independent prognostic factors for recurrence and survival in patients with endometrial cancer. We have also provided evidence that the use of a panel of biologically relevant immunomarkers, instead of single markers, is a tool that has the potential to identify high-risk patients or determine prognosis and subsequently guide therapeutic decision-making. Certainly, future prospective studies with integration of immunohistochemical profiling are necessary to validate this hypothesis in a larger series of endometrial cancer patients.

## Supporting information

S1 Table Staining and evaluation methods for all antibodies. (PDF)

S2 Table Characteristics of type I and type II tumors. (PDF)

S3 Table Associations between IHC markers and clinicopathological characteristics. (PDF)

S4 Table Associations among the IHC markers. (PDF)

S5 Table Follow-up time, overall survival (OS) and mortality rates during follow-up. (PDF)

S6 Table Hazard ratios (95% CI) estimated from univariate Cox regression analyses for each of the clinicopathological characteristics and IHC markers in the entire cohort, N = 204. (PDF)

S7 Table Hazard ratios (95% CIs) estimated from multivariate Cox regression analyses for each IHC marker adjusted for endometrial cancer type, grade, stage and depth of invasion. (PDF)

S8 Table Associations between cluster membership, IHC markers and clinicopathological characteristics. (PDF)

S1 Fig Representative examples of positive and negative classical IHC markers in endometrial carcinomas. (PDF)

S2 Fig Median marker values by cluster membership. (PDF)
