# Supplementary material for: Mismatch repair deficiency and aberrations in the Notch and Hedgehog pathways are of prognostic value in patients with endometrial cancer
Source: PLoS One. 2018 Dec 6;13(12):e0208221. doi: 10.1371/journal.pone.0208221 (PMC6283658; doi:10.1371/journal.pone.0208221)
Supplement: S1 Table — (PDF) [file pone.0208221.s001.pdf]

S1 Table: Staining and evaluation methods for all antibodies

IHC ABS	CLONE/CODE	MANUFACTURER	STAINING PROTOCOL	EVALUATION OF IHC/PROPOSED CUTOFFS	REFERENCES
**ER**	Clone 6F11, code NCL-L-ER-6F11	Novocastra, Leica Biosystems	20'ER1, 1:70-20', Bond Polymers Kit	positive: Allred: ≥3 (S+P)=0-8	J Clin Oncol. 1999 May;17(5):1474-81.
**PgR**	Clone 1A6, code NCL-L-PGR	Novocastra, Leica Biosystems	20'ER1, 1:70-20', Bond Polymers Kit	positive: Allred: ≥3 (S+P)=0-8	J Clin Oncol. 1999 May;17(5):1474-81.
**HER2**	Code A 0485	DAKO	20'ER1, 1:500-30', Bond Polymers Kit	1+ to 3+, 2+=equivocal, FISH performed, 3+=positive	J Clin Oncol. 2007 Jan 1;25(1):118-45
**p53**	Clone DO-7, code M7001	DAKO	20'ER1, Ab1:100-20', Bond polymers	any intensity in >10% of tumor cells stained considered as positive	Clin Cancer Res. 2011 Aug 1;17(15):5170-8
**Notch1**	Code ab8925, polyclonal	Abcam	20' ER1,10'PB, 1:200- 30', 30' ENVISION	0=0 to 10%, 1=11 to 25%, 2=26% to 50%, 3=51% to 75%, 4=>75%, IRS: SxP=0-12, ROC	Anticancer Res. 2014 Nov;34(11):6495-503
**Notch2**	Code ab72803, polyclonal	Abcam	20' ER2, 1:100- 30', Bond Polymers Kit	0=0 to 10%, 1=11 to 25%, 2=26% to 50%, 3=51% to 75%, 4=>75%, IRS: SxP=0-12, ROC	Anticancer Res. 2014 Nov;34(11):6495-503
**Notch3**	Code ab23426, polyclonal	Abcam	20' ER1, 1:500- 30', Bond Polymers Kit	0=0 to 10%, 1=11to 25%, 2=26% to 50%, 3=51% to 75%, 4=>75%, IRS: SxP=0-12, ROC	Anticancer Res. 2014 Nov;34(11):6495-503
**Jag1**	Clone hJ1(CD339), Code:HPA021555	Sigmaaldrich	20'ER1, 1:125-O/N, Bond Polymers Kit	0=0 to 10%, 1=11to 25%, 2=26% to 50%, 3=51% to 75%, 4=>75%, IRS: SxP=0-12, ROC	Anticancer Res. 2014 Nov;34(11):6495-503
**Sonic**	Code NBP1-36497, polyclonal	Novus Biologicals	20'ER2, 1:100-30', Bond Polymers Kit	0=0 to 5%, 1=>5 to 25%, 2=26% to 50%, 3=51% to 75%, 4=>75%, IRS: SxP=0-12 +:>3, ROC	Modern Pathology (2006) 19, 1139–1147
**Gli-1**	Code AF3324, polyclonal	R&D Biosystems	25'ER1, 1:50-1h, antigoat-25', Bond Polymers Kit	0=0 to 5%, 1=>5 to 25%, 2=26% to 50%, 3=51% to 75%, 4=>75%, IRS: SxP=0-12 +:>3, ROC	Modern Pathology (2006) 19, 1139–1147
**Smo**	Clone 3D10, Code LS-B6399	LSBio(LifeSpanBiosciences)	20'ER2, 1:600-O/N, 30' ENVISION	0=0 to 5%, 1=>5 to 25%, 2=26% to 50%, 3=51% to 75%, 4=>75%, IRS: SxP=0-12 +:>3, ROC	Modern Pathology (2006) 19, 1139–1147
**Patched-1**	Clone 5c7, Code NBP1-47945	Novus Biologicals	20'ER2, 1:150-30', Bond Polymers Kit	0=0 to 5%, 1=>5 to 25%, 2=26% to 50%, 3=51% to 75%, 4=>75%, IRS: SxP=0-12 +:>3, ROC	Modern Pathology (2006) 19, 1139–1147
**p16**	CINtec histology kit, code 9511, cloneE6H4	mtm Laboratories AG, ROCHE	20'ER2, RTU, Roche Kit	positive staining >25%	J Oncol. 2009;2009:305908. doi: 10.1155/2009/305908
**MLH 1**	Clone ES05, Cat No MONX11100	MONOSAN	20'ER1, Ab 1:60-30', Bond Polymers Kit	any intensity in ≥10% of tumor cells stained considered as positive	Clin Cancer Res 2008;14(6)March15, 2008 & PMID 24503759
**MSH 6**	Clone EP49, code M3646	DAKO	20'ER2, Ab 1:70-30', Bond Polymers Kit	any intensity in ≥10% of tumor cells stained considered as positive	Clin Cancer Res 2008;14(6)March15, 2008 & PMID 24503759
**PMS2**	clone M0R4G, NCL-L-PMS2	Novocastra, Leica Biosystems	20'ER2, Ab 1:60-30', Bond Polymers Kit	any intensity in ≥10% of tumor cells stained considered as positive	Clin Cancer Res 2008;14(6)March15, 2008 & PMID 24503759
**MSH 2**	Clone 25D12, Code NCL-MSH2	Novocastra, Leica Biosystems	20'ER1, Ab 1:30-30', Bond Polymers Kit	any intensity in ≥10% of tumor cells stained considered as positive	Clin Cancer Res 2008;14(6)March15, 2008 & PMID 24503759
