# Supplementary material for: Mismatch repair deficiency and aberrations in the Notch and Hedgehog pathways are of prognostic value in patients with endometrial cancer
Source: PLoS One. 2018 Dec 6;13(12):e0208221. doi: 10.1371/journal.pone.0208221 (PMC6283658; doi:10.1371/journal.pone.0208221)
Supplement: S2 Table — (PDF) [file pone.0208221.s002.pdf]

S2 Table: Characteristics of type I and type II tumors. Numbers in parenetheses: column %

	Type I (N=164)	N (%)	Type II (N=37)	N (%)
**Histology**	endometrioid carcinoma^	164 (100%)	endometrioid carcinoma	0 (0%)
			serous papillary or clear cell carcinoı	22 (59.5%)
			mixed carcinoma	8 (21.6%)
			other*	7 (18.9%)
**Age**	Median	63.1	Median	66.5
	Range	29-87	Range	33-79
**Stage**	I	117 (71.3%)	I	16 (43.3%)
	II	11 (6.7%)	II	2 (5.4%)
	III	26 (15.9)	III	10 (27%)
	IV	4 (2.4%)	IV	9 (24.3%)
	Unknown	6 (3.7%)	Unknown	0
**Stage**	Early	131 (79.9%)	Early	13 (35.1%)
	Advanced	33 (20.1%)	Advanced	24 (64.9%)
**Grade**	1	52 (31.7%)	1	1 (2.7%)
	2	84(51.2%)	2	4 (10.8%)
	3	28 (17.1%)	3	28 (75.7%)
	Unknown	0	Unknown	4 (10.8%)
**ER**	positive	109 (66.4%)	positive	17 (45.9%)
	negative	49 (29.9%)	negative	15 (40.6%)
	Unknown	6 (3.7%)	Unknown	5 (13.5%)
**PgR**	positive	121 (73,8%)	positive	14 (37.8%)
	negative	35 (21.3%)	negative	18 (48.7%)
	Unknown	8 (4.9%)	Unknown	5 (13.5%)
**p53**	overexpression	29 (17.7%)	overexpression	18 (48.7%)
	no overexpression	129 (78.6%)	No overexpression	14 (37.8%)
	Unknown	6 (3.7%)	Unknown	5 (13.5%)
**MMR**	proficient	73 (44.5%)	proficient	23 (62.2%)
	deficient	75 (45.7%)	deficient	6 (16.2%)
	Unknown	16 (9.8%)	Unknown	8 (21.6%)
**HER2**	positive	53 (32.3%)	positive	12 (32.4%)
	negative	105 (64.0%)	negative	18 (48.7%)
	Unknown	6 (3.7%)	Unknown	7 (18.9%)
**Ki67**	High (≥20)	92 (56.1%)	High (≥20)	24 (64.9%)
	Low (<20)	65 (39.6%)	Low (<20)	5 (13.5%0
	Unknown	7 (4.3%)	Unknown	8 (21.6%)
**PTEN**	Loss	105 (64.0%)	Loss	11 (29.7%)
	no loss	50 (30.5%)	no loss	21 (56.8%)
	Unknown	9 (5.5%)	Unknown	5 (13.5%)
**p16**	positive	61 (37.2%)	positive	25 (67.6%)
	negative	92 (56.1%)	negative	4 (10.8%)
	Unknown	11 (6.7%)	Unknown	8 (21.6%)
**Jag1**	positive	44 (26.8%)	positive	11 (29.7%)
	negative	113 (68.9%)	negative	20 (54.1%)
	Unknown	7 (4.3%)	Unknown	6 (16.2%)
**Notch2**	positive	22 (13.4%)	positive	15 (40.5%)
	negative	135 (82.3%)	negative	17 (46.0%)
	Unknown	7 (4.3%)	Unknown	5 (13.5%)
**Notch3**	positive	15 (9.2%)	positive	9 (24.3%)
	negative	142 (86.6%)	negative	23 (62.2%)
	Unknown	7 (4.3%)	Unknown	5 (13.5%)
**Gli**	positive	53 (32.3%)	positive	6 (16.2%)
	negative	103 (62.8%)	negative	27 (73.0%)
	Unknown	8 (4.9%)	Unknown	4 (10.8%)
**Patched-1**	positive	150 (91.5%)	positive	30 (81.1%)
	negative	4 (2.4%)	negative	0 (0%)
	Unknown	10 (6.1%)	Unknown	7 (18.9%)
**Ssh**	positive	150 (91.5%)	positive	30 (81.1%)
	negative	4 (2.4%)	negative	0 (0%)
	Unknown	10 (6.1%)	Unknown	7 (18.9%)
**Smo**	positive	56 (34.2%)	positive	15 (40.5%)
	negative	95 (57.9%)	negative	16 (43.2%)
	Unknown	13 (7.9%)	Unknown	6 (16.2%)

^ : 3 carcinomas with squamous differentiation; * : 3 carcinosarcomas, 1 undifferentiated carcinoma, 2 poorly differentiated endometrioid carcinoma up to >90% with serous foci, 1 unspecified adenocarcinoma
