# Supplementary material for: Mismatch repair deficiency and aberrations in the Notch and Hedgehog pathways are of prognostic value in patients with endometrial cancer
Source: PLoS One. 2018 Dec 6;13(12):e0208221. doi: 10.1371/journal.pone.0208221 (PMC6283658; doi:10.1371/journal.pone.0208221)
Supplement: S3 Table — (PDF) [file pone.0208221.s003.pdf]

S3 Table: Associations between IHC markers and clinicopathological characteristics. Numbers in parenetheses: %

		Age (median cut-off)				Type				Stage				Grade					Depth of invasion			
		N	≤64	>64	p-value	N	I	II	p-value	N	I-II	III-IV	p-value	N	1	2	3	p-value	N	<50%	>50%	p-value
**ER status**	Negative	64	27 (28.1)	37 (38.9)	0.11	64	49 (31.0)	15 (46.9)	0.083	64	48 (34.3)	16 (35.6)	0.88	62	14 (26.9)	34 (40.0)	14 (28.0)	0.19	64	26 (28.3)	38 (41.3)	0.063
	Positive	127	69 (71.9)	58 (61.1)		126	109 (69.0)	17 (53.1)		121	92 (65.7)	29 (64.4)		125	38 (73.1)	51 (60.0)	36 (72.0)		120	66 (71.7)	54 (58.7)	
**PgR status**	Negative	53	26 (27.4)	27 (28.7)	0.84	53	35 (22.4)	18 (56.3)	**<0.001**	53	31 (22.5)	22 (48.9)	**0.001**	42	10 (19.6)	19 (22.6)	22 (44.0)	**0.009**	53	20 (22.0)	33 (36.3)	**0.034**
	Positive	136	69 (72.6)	67 (71.3)		135	121 (77.6)	14 (43.8)		130	107 (77.5)	23 (51.1)		134	41 (80.4)	65 (77.4)	28 (56.0)		129	71 (78.0)	58 (63.7)	
**HER2 status**	Negative	124	64 (66.7)	60 (64.5)	0.76	123	105 (66.5)	18 (60.0)	0.5	123	94 (68.1)	29 (64.4)	0.65	124	34 (65.4)	58 (68.2)	32 (65.3)	0.92	123	68 (74.7)	55 (60.4)	**0.04**
	Positive	65	32 (33.3)	33 (35.5)		65	53 (33.5)	12 (40.0)		60	44 (31.9)	16 (35.6)		62	18 (34.6)	27 (31.8)	17 (34.7)		59	23 (25.3)	36 (39.6)	
**Ki67 status**	High	117	60 (63.8)	57 (61.3)	0.72	116	92 (58.6)	24 (82.8)	**0.014**	112	77 (56.2)	35 (79.5)	**0.006**	114	22 (43.1)	50 (59.5)	42 (85.7)	**<0.001**	111	57 (64.0)	54 (59.3)	0.52
	Low	70	34 (36.2)	36 (38.7)		70	65 (41.4)	5 (17.2)		69	60 (43.8)	9 (20.5)		70	29 (56.9)	34 (40.5)	7 (14.3)		69	32 (36.0)	37 (40.7)	
**p53**	Overexpression	48	15 (15.6)	33 (34.7)	**0.002**	47	29 (18.4)	18 (56.3)	**<0.001**	45	26 (18.6)	19 (42.2)	**0.001**	46	5 (9.6)	14 (16.5)	27 (54.0)	**<0.001**	45	19 (20.7)	26 (28.3)	0.23
	No overexpression	143	81 (84.4)	62 (65.3)		143	129 (81.6)	14 (43.8)		140	114 (81.4)	26 (57.8)		141	47 (90.4)	71 (83.5)	23 (46.0)		139	73 (79.3)	66 (71.7)	
**p16**	Negative	97	52 (57.1)	45 (48.9)	0.27	96	92 (60.1)	4 (13.8)	**<0.001**	93	80 (59.3)	13 (31.0)	**0.001**	96	35 (70.0)	50 (61.7)	11 (22.4)	**<0.001**	92	47 (54.0)	45 (50.6)	0.65
	Positive	86	39 (42.9)	47 (51.1)		86	61 (39.9)	25 (86.2)		84	55 (40.7)	29 (69.0)		84	15 (30.0)	31 (38.3)	38 (77.6)		84	40 (46.0)	44 (49.4)	
**PTEN status**	Loss	117	61 (64.2)	56 (60.2)	0.57	116	105 (67.7)	11 (34.4)	**<0.001**	113	89 (65.0)	24 (53.3)	0.16	116	34 (68.0)	62 (73.8)	20 (40.0)	**<0.001**	113	60 (65.9)	53 (58.9)	0.33
	No loss	71	34 (35.8)	37 (39.8)		71	50 (32.3)	21 (65.6)		69	48 (35.0)	21 (46.7)		68	16 (32.0)	22 (26.2)	30 (60.0)		68	31 (34.1)	37 (41.1)	
**Jag1**	Negative (0-4)	134	64 (67.4)	70 (74.5)	0.28	133	113 (72.0)	20 (64.5)	0.4	129	98 (70.5)	31 (70.5)	0.99	132	35 (67.3)	66 (78.6)	31 (63.3)	0.13	128	61 (67.8)	67 (72.8)	0.46
	Positive(5-9)	55	31 (32.6)	24 (25.5)		55	44 (28.0)	11 (35.5)		54	41 (29.5)	13 (29.5)		53	17 (32.7)	18 (21.4)	18 (36.7)		54	29 (32.2)	25 (27.2)	
**Notch2 status**	Negative (0-4)	153	74 (77.1)	79 (84.0)	0.23	152	135 (86.0)	17 (53.1)	**<0.001**	147	121 (87.1)	26 (57.8)	**<0.001**	151	49 (94.2)	72 (85.7)	30 (60.0)	**<0.001**	146	74 (80.4)	72 (79.1)	0.83
	Positive(5-9)	37	22 (22.9)	15 (16.0)		37	22 (14.0)	15 (46.9)		37	18 (12.9)	19 (42.2)		35	3 (5.8)	12 (14.3)	20 (40.0)		37	18 (19.6)	19 (20.9)	
**Notch3 status**	Negative (0-4)	166	82 (86.3)	84 (88.4)	0.66	165	142 (90.4)	23 (71.9)	**0.004**	160	129 (92.1)	31 (70.5)	**<0.001**	164	50 (96.2)	79 (94.0)	35 (70.0)	**<0.001**	159	82 (89.1)	77 (84.6)	0.37
	Positive(5-9)	24	13 (13.7)	11 (11.6)		24	15 (9.6)	9 (28.1)		24	11 (7.9)	13 (29.5)		22	2 (3.8)	5 (6.0)	15 (30.0)		24	10 (10.9)	14 (15.4)	
**Glli**	Negative (0-2)	131	66 (68.8)	65 (69.1)	0.95	130	103 (66.0)	27 (81.8)	0.075	126	91 (65.9)	35 (76.1)	0.2	128	33 (63.5)	55 (66.3)	40 (78.4)	0.21	126	61 (67.0)	65 (70.7)	0.6
	Positive (3-12)	59	30 (31.3)	29 (30.9)		59	53 (34.0)	6 (18.2)		58	47 (34.1)	11 (23.9)		58	19 (36.5)	28 (33.7)	11 (21.6)		57	30 (33.0)	27 (29.3)	
**Patched-1**	Negative (0-2)	126	63 (66.3)	63 (67.7)	0.84	125	108 (69.2)	17 (54.8)	0.12	124	95 (69.3)	29 (64.4)	0.54	125	36 (69.2)	62 (74.7)	27 (54.0)	**0.045**	123	60 (65.9)	63 (70.0)	0.56
	Positive (3-12)	62	32 (33.7)	30 (32.3)		62	48 (30.8)	14 (45.2)		58	42 (30.7)	16 (35.6)		60	16 (30.8)	21 (25.3)	23 (46.0)		58	31 (34.1)	27 (30.0)	
**Shh**	Negative (0-2)	5	1 (1.1)	4 (4.3)	0.17	4	4 (2.6)	0 (0)	0.37	5	5 (3.7)	0 (0)	0.2	5	4 (7.8)	0 (0)	1 (2.0)	**0.025**	5	3 (3.4)	2 (2.2)	0.63
	Positive (3-12)	180	92 (98.9)	88 (95.7)		180	150 (97.4)	30 (100.0)		174	130 (96.3)	44 (100.0)		177	47 (92.2)	82 (100.0)	48 (98.0)		173	85 (96.6)	88 (97.8)	
**Smo**	Negative (0-2)	112	57 (63.3)	55 (59.1)	0.56	111	95 (62.9)	16 (51.6)	0.24	109	78 (58.6)	31 (70.5)	0.16	110	33 (66.0)	50 (62.5)	27 (54.0)	0.44	109	53 (60.2)	56 (63.6)	0.64
	Positive (3-12)	71	33 (36.7)	38 (40.9)		71	56 (37.1)	15 (48.4)		68	55 (41.4)	13 (29.5)		70	17 (34.0)	30 (37.5)	23 (46.0)		67	35 (39.8)	32 (36.4)	
**MMR status**	Deficiency	81	38 (43.7)	43 (47.3)	0.63	81	75 (50.7)	6 (20.7)	**0.003**	78	65 (50.0)	13 (31.0)	**0.031**	81	31 (64.6)	33 (42.9)	17 (34.0)	**0.007**	78	39 (45.9)	39 (45.3)	0.94
	Proficiency	97	49 (56.3)	48 (52.7)		96	73 (49.3)	23 (79.3)		94	65 (50.0)	29 (69.0)		94	17 (35.4)	44 (57.1)	33 (66.0)		93	46 (54.1)	47 (54.7)	
