# Supplementary material for: Mismatch repair deficiency and aberrations in the Notch and Hedgehog pathways are of prognostic value in patients with endometrial cancer
Source: PLoS One. 2018 Dec 6;13(12):e0208221. doi: 10.1371/journal.pone.0208221 (PMC6283658; doi:10.1371/journal.pone.0208221)
Supplement: S4 Table — (PDF) [file pone.0208221.s004.pdf]

S4 Table: Associations among the IHC markers. Numbers in parenetheses: %

		JAG1 final status			NOTCH2 final status			NOTCH3 final status			GLI (cutoff at 3)			PATCH (cutoff at 3)			Shh (cutoff at 3)			Smo (cutoff at 3)		
		**Negative (0-4)**	**Positive(5-9)**	**p-value**	**Negative (0-4)**	**Positive(5-9)**	**p-value**	**Negative (0-4)**	**Positive(5-9)**	**p-value**	**Negative**	**Positive**	**p-value**	**Negative**	**Positive**	**p-value**	**Negative**	**Positive**	**p-value**	**Negative**	**Positive**	**p-value**
ER status	Negative	51 (38.1)	12 (21.8)	**0.041**	51 (33.3)	12 (32.4)	>0.999	56 (33.7)	7 (29.2)	0.817	50 (38.5)	13 (22.0)	**0.031**	52 (41.3)	11 (17.7)	**0.002**	4 (80.0)	57 (31.7)	**0.041**	45 (40.2)	15 (21.1)	**0.01**
	Positive	83 (61.9)	43 (78.2)		102 (66.7)	25 (67.6)		110 (66.3)	17 (70.8)		80 (61.5)	46 (78.0)		74 (58.7)	51 (82.3)		1 (20.0)	123 (68.3)		67 (59.8)	56 (78.9)	
PgR status	Negative	38 (28.8)	14 (25.5)	0.722	39 (25.8)	14 (37.8)	0.157	40 (24.4)	12 (50.0)	**0.014**	43 (33.1)	10 (17.2)	**0.035**	43 (34.4)	10 (16.1)	**0.01**	2 (40.0)	50 (27.9)	0.622	38 (33.9)	15 (21.1)	0.068
	Positive	94 (71.2)	41 (74.5)		112 (74.2)	23 (62.2)		124 (75.6)	12 (50.0)		87 (66.9)	48 (82.8)		82 (65.6)	52 (83.9)		3 (60.0)	129 (72.1)		74 (66.1)	56 (78.9)	
HER2 status	Negative	89 (66.9)	34 (61.8)	0.506	93 (61.6)	30 (81.1)	**0.033**	105 (64.0)	18 (75.0)	0.362	88 (68.2)	34 (58.6)	0.245	87 (69.6)	35 (56.5)	0.102	5 (100.0)	115 (63.9)	0.164	78 (70.3)	40 (56.3)	0.059
	Positive	44 (33.1)	21 (38.2)		58 (38.4)	7 (18.9)		59 (36.0)	6 (25.0)		41 (31.8)	24 (41.4)		38 (30.4)	27 (43.5)		0 (0)	65 (36.1)		33 (29.7)	31 (43.7)	
Ki67 status	High	84 (63.6)	33 (60.0)	0.74	84 (56.0)	32 (88.9)	**<0.001**	93 (57.4)	24 (100.0)	**<0.001**	88 (69.3)	27 (46.6)	**0.005**	65 (52.4)	50 (82.0)	**<0.001**	1 (20.0)	114 (63.7)	0.067	56 (50.9)	59 (83.1)	**<0.001**
	Low	48 (36.4)	22 (40.0)		66 (44.0)	4 (11.1)		69 (42.6)			39 (30.7)	31 (53.4)		59 (47.6)	11 (18.0)		4 (80.0)	65 (36.3)		54 (49.1)	12 (16.9)	
P53 status (75% cutoff)	Overexpression	32 (23.9)	16 (29.1)	0.466	32 (20.9)	16 (43.2)	0.01	36 (21.7)	12 (50.0)	**0.005**	37 (28.5)	10 (16.9)	0.104	23 (18.3)	24 (38.7)	**0.004**	1 (20.0)	46 (25.6)	>0.999	23 (20.5)	24 (33.8)	0.056
	No overexpression	102 (76.1)	39 (70.9)		121 (79.1)	21 (56.8)		130 (78.3)	12 (50.0)		93 (71.5)	49 (83.1)		103 (81.7)	38 (61.3)		4 (80.0)	134 (74.4)		89 (79.5)	47 (66.2)	
p16 status	Negative	73 (56.6)	24 (44.4)	0.146	92 (62.6)	4 (11.4)	**<0.001**	92 (57.9)	5 (20.8)	**0.001**	62 (49.6)	34 (59.6)	0.263	73 (60.3)	23 (37.7)	**0.005**	5 (100.0)	90 (51.1)	0.061	60 (56.1)	33 (46.5)	0.224
	Positive	56 (43.4)	30 (55.6)		55 (37.4)	31 (88.6)		67 (42.1)	19 (79.2)		63 (50.4)	23 (40.4)		48 (39.7)	38 (62.3)		0 (0)	86 (48.9)		47 (43.9)	38 (53.5)	
PTEN status	loss	86 (66.2)	31 (56.4)	0.244	100 (67.1)	16 (43.2)	0.013	110 (67.9)	7 (29.2)	**<0.001**	78 (60.9)	38 (66.7)	0.512	85 (68.5)	31 (51.7)	**0.034**	4 (80.0)	111 (63.1)	0.654	73 (66.4)	41 (58.6)	0.342
	no loss	44 (33.8)	24 (43.6)		49 (32.9)	21 (56.8)		52 (32.1)	17 (70.8)		50 (39.1)	19 (33.3)		39 (31.5)	29 (48.3)		1 (20.0)	65 (36.9)		37 (33.6)	29 (41.4)	
Jag1 status	Negative (0-4)	134 (100.0)		-	109 (71.7)	24 (66.7)	0.547	116 (70.7)	17 (70.8)	>0.999	90 (70.3)	42 (71.2)	>0.999	95 (76.0)	36 (59.0)	**0.025**	3 (60.0)	127 (70.6)	0.635	76 (68.5)	52 (73.2)	0.511
	Positive(5-9)		55 (100.0)		43 (28.3)	12 (33.3)		48 (29.3)	7 (29.2)		38 (29.7)	17 (28.8)		30 (24.0)	25 (41.0)		2 (40.0)	53 (29.4)		35 (31.5)	19 (26.8)	
Notch2 status	Negative (0-4)	109 (82.0)	43 (78.2)	0.547	153 (100.0)		-	147 (89.1)	6 (25.0)	**<0.001**	98 (76.0)	53 (89.8)	**0.03**	106 (84.8)	44 (71.0)	**0.032**	5 (100.0)	143 (79.9)	0.585	90 (80.4)	56 (80.0)	>0.999
	Positive(5-9)	24 (18.0)	12 (21.8)			37 (100.0)		18 (10.9)	18 (75.0)		31 (24.0)	6 (10.2)		19 (15.2)	18 (29.0)		0 (0)	36 (20.1)		22 (19.6)	14 (20.0)	
Notch3 status	Negative (0-4)	116 (87.2)	48 (87.3)	>0.999	147 (96.1)	18 (50.0)	**<0.001**	166 (100.0)		-	108 (83.7)	56 (94.9)	**0.035**	110 (88.0)	53 (85.5)	0.647	5 (100.0)	155 (86.6)	>0.999	94 (84.7)	64 (90.1)	0.371
	Positive(5-9)	17 (12.8)	7 (12.7)		6 (3.9)	18 (50.0)			24 (100.0)		21 (16.3)	3 (5.1)		15 (12.0)	9 (14.5)		0 (0)	24 (13.4)		17 (15.3)	7 (9.9)	
Gli (cutoff at 3)	Negative	90 (68.2)	38 (69.1)	>0.999	98 (64.9)	**31 (83.8)**	**0.03**	108 (65.9)	**21 (87.5)**	**0.035**	131 (100.0)		-	87 (69.0)	43 (69.4)	>0.999	5 (100.0)	122 (67.8)	0.327	77 (68.8)	50 (70.4)	0.87
	Positive	42 (31.8)	17 (30.9)		53 (35.1)	6 (16.2)		56 (34.1)	3 (12.5)			59 (100.0)		39 (31.0)	19 (30.6)		0 (0)	58 (32.2)		35 (31.3)	21 (29.6)	
Patched-1 (cutoff at 3)	Negative	95 (72.5)	**30 (54.5)**	**0.025**	106 (70.7)	19 (51.4)	0.032	110 (67.5)	15 (62.5)	0.647	87 (66.9)	39 (67.2)	>0.999	126 (100.0)		-	4 (80.0)	120 (66.7)	>0.999	84 (75.0)	39 (54.9)	**0.006**
	Positive	36 (27.5)	25 (45.5)		44 (29.3)	18 (48.6)		53 (32.5)	9 (37.5)		43 (33.1)	19 (32.8)			62 (100.0)		1 (20.0)	60 (33.3)		28 (25.0)	32 (45.1)	
Shh (cutoff at 3)	Negative	3 (2.3)	2 (3.6)	0.635	5 (3.4)	0 (0)	0.585	5 (3.1)	0 (0)	>0.999	5 (3.9)	0 (0)	0.327	4 (3.2)	1 (1.6)	>0.999	5 (100.0)		-	5 (4.5)	0 (0)	0.158
	Positive	127 (97.7)	53 (96.4)		143 (96.6)	36 (100.0)		155 (96.9)	24 (100.0)		122 (96.1)	58 (100.0)		120 (96.8)	60 (98.4)			180 (100.0)		106 (95.5)	71 (100.0)	
Smo (cutoff at 3)	Negative	76 (59.4)	35 (64.8)	0.511	90 (61.6)	22 (61.1)	>0.999	94 (59.5)	17 (70.8)	0.371	77 (60.6)	35 (62.5)	0.87	84 (68.3)	28 (46.7)	**0.006**	5 (100.0)	106 (59.9)	0.158	112 (100.0)		-
	Positive	52 (40.6)	19 (35.2)		56 (38.4)	14 (38.9)		64 (40.5)	7 (29.2)		50 (39.4)	21 (37.5)		39 (31.7)	32 (53.3)		0 (0)	71 (40.1)			71 (100.0)	
MMR status	def	61 (49.2)	20 (39.2)	0.247	71 (50.0)	9 (26.5)	**0.02**	73 (48.0)	8 (33.3)	0.195	60 (48.8)	21 (39.6)	0.323	61 (53.0)	20 (32.8)	**0.011**	4 (80.0)	76 (45.2)	0.183	50 (49.0)	29 (41.4)	0.353
	prof	63 (50.8)	31 (60.8)		71 (50.0)	25 (73.5)		79 (52.0)	16 (66.7)		63 (51.2)	32 (60.4)		54 (47.0)	41 (67.2)		1 (20.0)	92 (54.8)		52 (51.0)	41 (58.6)	
