# Supplementary material for: Mismatch repair deficiency and aberrations in the Notch and Hedgehog pathways are of prognostic value in patients with endometrial cancer
Source: PLoS One. 2018 Dec 6;13(12):e0208221. doi: 10.1371/journal.pone.0208221 (PMC6283658; doi:10.1371/journal.pone.0208221)
Supplement: S5 Table — (PDF) [file pone.0208221.s005.pdf]

S5 Table: Follow-up time, overall survival (OS) and mortality rates during follow-up.

	Entire cohort (N=204)
**OS (months)**	
Median	225
N (%) deaths	59 (28.9%)
**5-year OS (months)**	
Median	Not reached
N (%) deaths	52 (25.5%)
**DFS (months)**	
Median	133.8
N (%) relapses	45 (22.1%)
**DFS 5-year (months)**	
Median	Not reached
N (%) relapses	42 (20.6%)
**Follow-up period (months)**	
Median	71.7
Mean	58.2
Range	1-236
