# Supplementary material for: Mismatch repair deficiency and aberrations in the Notch and Hedgehog pathways are of prognostic value in patients with endometrial cancer
Source: PLoS One. 2018 Dec 6;13(12):e0208221. doi: 10.1371/journal.pone.0208221 (PMC6283658; doi:10.1371/journal.pone.0208221)
Supplement: S6 Table — (PDF) [file pone.0208221.s006.pdf]

S6 Table: Hazard ratios (95% CI) estimated from univariate Cox regression analyses for each of the clinicopathological characteristics and IHC markers in the entire cohort, N=204.

Parameter	Categories	N patients	N events	HR	95% CI	Wald's p	
**5- year DFS**							
	Clinicopathological & treatment parameters						
Age (median cut-off)	>64 vs. ≤64	99 vs. 105	19 vs. 23	0.89	0.49-1.64	0.72	
Type	II vs. I	37 vs. 164	14 vs. 28	3.01	1.57-5.76	**0.001**	
Grade	1 vs. 3	53 vs. 58	5 vs. 22	0.15	0.06-0.41	**<0.001**	
	2 vs. 3	89 vs. 58	13 vs. 22	0.27	0.13-0.53	**<0.001**	
Stage	III-IV vs. I-II	50 vs. 148	15 vs. 27	2.66	1.40-5.04	**0.003**	
Depth of invasion	>50% vs. <50%	99 vs. 97	27 vs. 15	2.08	1.10-3.91	**0.023**	
Adjuvant chemotherapy	Yes vs. No	24 vs. 178	7 vs. 35	1.42	0.63-3.21	0.4	
Adjuvant radiotherapy	Yes vs. No	121 vs. 82	30 vs. 12	1.62	0.83-3.16	0.16	
	IHC markers						
ER status	Positive vs. Negative	127 vs. 64	23 vs. 16	0.72	0.38-1.37	0.32	
PgR status	Positive vs. Negative	136 vs. 53	25 vs. 14	0.61	0.32-1.18	0.14	
HER2 status	Positive vs. Negative	65 vs. 124	12 vs. 26	0.89	0.45-1.76	0.74	
p53 status (75% cutoff)	Overexpression vs, No overexpression	48 vs. 143	12 vs. 27	1.71	0.86-3.38	0.13	
p16 status	Positive vs. Negative	86 vs. 97	23 vs. 13	2.46	1.24-4.87	**0.01**	
Ki67 status	High vs. Low	117 vs. 70	28 vs. 9	2.17	1.02-4.60	**0.044**	
PTEN status	No loss vs. loss	71 vs. 117	22 vs. 15	2.87	1.49-5.54	**0.002**	
Jag1 status	Positive(5-9) vs. Negative(0-4)	55 vs. 134	10 vs. 29	0.73	0.35-1.49	0.38	
Notch2 status	Positive(5-9) vs. Negative(0-4)	37 vs. 153	15 vs. 24	3.67	1.90-7.10	**<0.001**	
Notch3 status	Positive(5-9) vs. Negative(0-4)	24 vs. 166	8 vs. 30	2.6	1.18-5.72	**0.017**	
Gli (cutoff at 3)	Positive vs. Negative	59 vs. 131	10 vs. 29	0.7	0.34-1.44	0.33	
Patched-1 (cutoff at 3)	Positive vs. Negative	62 vs. 126	0 vs. 19	2.13	1.13-4.01	**0.02**	
Shh (cutoff at 3)	Positive vs. Negative	180 vs. 5	38 vs. 0	-	-	-	Cannot be estimated due to 0 events in reference category
Smo (cutoff at 3)	Positive vs. Negative	71 vs. 112	17 vs. 20	1.38	0.72-2.63	0.33	
MMR status	Proficiency vs. deficiency	97 vs. 81	25 vs. 8	3.02	1.36-6.72	**0.007**	
**5- year OS**							
	Clinicopathological & treatment parameters						
Age (median cut-off)	>64 vs. ≤64	99 vs. 105	27 vs. 25	1.18	0.68-2.03	0.56	
Type	II vs. I	37 vs. 164	17 vs. 33	3	1.66-5.41	**<0.001**	
Grade	1 vs. 3	53 vs. 58	5 vs. 29	0.12	0.05-0.31	**<0.001**	
	2 vs. 3	89 vs. 58	17 vs. 29	0.27	0.15-0.49	**<0.001**	
Stage	III-IV vs. I-II	50 vs. 148	24 vs. 26	4.5	2.56-7.90	**<0.001**	
Depth of invasion	>50% vs. <50%	99 vs. 97	29 vs. 20	1.64	0.93-2.90	**0.09**	
Adjuvant chemotherapy	Yes vs. No	24 vs. 178	5 vs. 47	0.83	0.33-2.08	0.69	
Adjuvant radiotherapy	Yes vs. No	121 vs. 82	29 vs. 23	0.77	0.45-1.34	0.36	
	IHC markers						
ER status	Positive vs. Negative	127 vs. 64	30 vs. 18	0.84	0.47-1.51	0.56	
PgR status	Positive vs. Negative	136 vs. 53	31 vs. 17	0.63	0.35-1.14	0.13	
HER2 status	Positive vs. Negative	65 vs. 124	17 vs. 31	1.13	0.63-2.04	0.68	
p53 status (75% cutoff)	Overexpression vs, No overexpression	48 vs. 143	20 vs. 28	2.83	1.59-5.05	**<0.001**	
p16 status	Positive vs. Negative	86 vs. 97	30 vs. 15	2.76	1.48-5.14	**0.001**	
Ki67 status	High vs. Low	117 vs. 70	34 vs. 13	1.8	0.95-3.42	**0.071**	
PTEN status	No loss vs. loss	71 vs. 117	25 vs. 23	2.14	1.21-3.78	**0.009**	
Jag1 status	Positive(5-9) vs. Negative(0-4)	55 vs. 134	11 vs. 37	0.63	0.32-1.23	0.17	
Notch2 status	Positive(5-9) vs. Negative(0-4)	37 vs. 153	17 vs. 31	3.15	1.72-5.74	**<0.001**	
Notch3 status	Positive(5-9) vs. Negative(0-4)	24 vs. 166	12 vs. 35	3.49	1.80-6.78	**<0.001**	
Gli (cutoff at 3)	Positive vs. Negative	59 vs. 131	13 vs. 35	0.77	0.41-1.46	0.43	
Patched-1 (cutoff at 3)	Positive vs. Negative	62 vs. 126	20 vs. 28	1.44	0.81-2.56	0.21	
Shh (cutoff at 3)	Positive vs. Negative	180 vs. 5	47 vs. 1	1.39	0.19-10.07	0.74	
Smo (cutoff at 3)	Positive vs. Negative	71 vs. 112	19 vs. 28	1.05	0.58-1.87	0.88	
MMR status	Proficiency vs. deficiency	97 vs. 81	32 vs. 12	2.53	1.30-4.92	**0.006**	
