# Supplementary material for: Mismatch repair deficiency and aberrations in the Notch and Hedgehog pathways are of prognostic value in patients with endometrial cancer
Source: PLoS One. 2018 Dec 6;13(12):e0208221. doi: 10.1371/journal.pone.0208221 (PMC6283658; doi:10.1371/journal.pone.0208221)
Supplement: S7 Table — (PDF) [file pone.0208221.s007.pdf]

S7 Table: Hazard ratios (95% CIs) estimated from multivariate Cox regression analyses for each IHC marker adjusted for endometrial cancer type, grade, stage and depth of invasion.

Parameter	Categories	HR	95% CI		Wald's p	
**5- year DFS**						
	IHC markers					
ER status	Positive vs. Negative	0.794	0.406	1.555	0.5	
PgR status	Positive vs. Negative	1.077	0.516	2.246	0.84	
HER2 status	Positive vs. Negative	0.897	0.434	1.855	0.77	
P53 status (75% cutoff)	Overexpression vs, No overexpression	0.788	0.351	1.773	0.57	
p16 status	Positive vs. Negative	1.189	0.532	2.655	0.67	
Ki67 status	High vs. Low	1.261	0.554	2.871	0.58	
PTEN status	No loss vs. Loss	1.732	0.837	3.584	0.14	
Jag1 status	Positive(5-9) vs. Negative(0-4)	0.501	0.231	1.089	**0.081**	
Notch2 status	Positive(5-9) vs. Negative(0-4)	1.926	0.897	4.134	**0.093**	
Notch3 status	Positive(5-9) vs. Negative(0-4)	1.007	0.405	2.505	0.99	
Gli (cutoff at 3)	Positive vs. Negative	0.834	0.386	1.803	0.64	
Patched-1 (cutoff at 3)	Positive vs. Negative	2.037	1.047	3.964	**0.036**	
Shh (cutoff at 3)	Positive vs. Negative	-	-	-	-	Cannot be estimated due to 0 events in reference category
Smo (cutoff at 3)	Positive vs. Negative	1.513	0.769	2.98	0.23	
MMR status	Proficiency vs. deficiency	2.261	0.988	5.176	**0.054**	
**5- year OS**						
ER status	Positive vs. Negative	0.842	0.452	1.571	0.59	
PgR status	Positive vs. Negative	1.393	0.686	2.828	0.36	
HER2 status	Positive vs. Negative	1.19	0.619	2.291	0.6	
P53 status (75% cutoff)	Overexpression vs, No overexpression	1.299	0.642	2.629	0.47	
p16 status	Positive vs. Negative	1.719	0.803	3.683	0.16	
Ki67 status	High vs. Low	0.775	0.376	1.597	0.49	
Ki67 status (continuous)		1.468	0.762	2.826	0.42	
PTEN status	No loss vs. Loss	1.646	0.87	3.116	0.13	
Jag1 status	Positive(5-9) vs. Negative(0-4)	0.476	0.233	0.973	**0.042**	
Notch2 status	Positive(5-9) vs. Negative(0-4)	1.54	0.784	3.023	0.21	
Notch3 status	Positive(5-9) vs. Negative(0-4)	1.515	0.707	3.246	0.29	
Gli (cutoff at 3)	Positive vs. Negative	1.232	0.612	2.479	0.56	
Patched-1 (cutoff at 3)	Positive vs. Negative	1.112	0.593	2.086	0.74	
Shh (cutoff at 3)	Positive vs. Negative	-	-	-	-	Cannot be estimated due to 0 events in reference category
Smo (cutoff at 3)	Positive vs. Negative	1.022	0.542	1.929	0.95	
MMR status	Proficiency vs. deficiency	2.193	1.051	4.576	**0.036**	
