# Supplementary material for: Mismatch repair deficiency and aberrations in the Notch and Hedgehog pathways are of prognostic value in patients with endometrial cancer
Source: PLoS One. 2018 Dec 6;13(12):e0208221. doi: 10.1371/journal.pone.0208221 (PMC6283658; doi:10.1371/journal.pone.0208221)
Supplement: S8 Table — (PDF) [file pone.0208221.s008.pdf]

S8 Table: Associations between cluster membership, IHC markers and clinicopathological characteristics.Numbers in parenetheses: %

		Jag1/Notch clusters				Gli/Patch/Smo clusters				
		1	2	3	p-value	A	B	C	D	p-value
					Marker parameteres					
ER status	Negative	12 (20.3)	31 (38.3)	18 (38.3)	0.052	13 (28.3)	5 (15.2)	8 (28.6)	**34 (44.7)**	**0.017**
	Positive	47 (79.7)	50 (61.7)	29 (61.7)		33 (71.7)	28 (84.8)	20 (71.4)	42 (55.3)	
PgR status	Negative	7 (11.9)	22 (27.8)	**22 (46.8)**	**<0.001**	11 (23.9)	6 (18.2)	6 (21.4)	30 (39.5)	0.063
	Positive	52 (88.1)	57 (72.2)	25 (53.2)		35 (76.1)	27 (81.8)	22 (78.6)	46 (60.5)	
HER2 status	Negative	32 (54.2)	53 (66.3)	36 (76.6)	0.054	29 (63.0)	17 (51.5)	19 (67.9)	53 (70.7)	0.277
	Positive	27 (45.8)	27 (33.8)	11 (23.4)		17 (37.0)	16 (48.5)	9 (32.1)	22 (29.3)	
Ki67 status	High	32 (54.2)	41 (51.3)	**43 (93.5)**	**<0.001**	37 (80.4)	27 (81.8)	8 (28.6)	43 (58.1)	**<0.001**
	Low	27 (45.8)	39 (48.8)	3 (6.5)		9 (19.6)	6 (18.2)	**20 (71.4)**	31 (41.9)	
p53 status (75% cutoff)	Overexpression	14 (23.7)	13 (16.0)	**21 (44.7)**	**0.002**	10 (21.7)	17 (51.5)	3 (10.7)	17 (22.4)	**0.001**
	No overexpression	45 (76.3)	68 (84.0)	26 (55.3)		36 (78.3)	16 (48.5)	**25 (89.3)**	59 (77.6)	
p16 final status	Negative	36 (62.1)	52 (66.7)	8 (17.4)	**<0.001**	22 (47.8)	11 (33.3)	18 (66.7)	42 (58.3)	**0.038**
	Positive	22 (37.9)	26 (33.3)	**38 (82.6)**		24 (52.2)	**22 (66.7)**	9 (33.3)	30 (41.7)	
PTEN status	Loss	41 (70.7)	55 (70.5)	20 (42.6)	**0.003**	30 (65.2)	13 (40.6)	21 (77.8)	50 (66.7)	**0.019**
	No loss	17 (29.3)	23 (29.5)	**27 (57.4)**		16 (34.8)	**19 (59.4)**	6 (22.2)	25 (33.3)	
Jag1 status	Negative (0-4)	22 (37.3)	77 (95.1)	33 (70.2)	**<0.001**	38 (82.6)	18 (54.5)	20 (71.4)	52 (69.3)	0.062
	Positive(5-9)	37 (62.7)	4 (4.9)	14 (29.8)		8 (17.4)	15 (45.5)	8 (28.6)	23 (30.7)	
Notch2 status	Negative (0-4)	58 (98.3)	81 (100.0)	13 (27.7)	**<0.001**	38 (84.4)	21 (63.6)	26 (92.9)	61 (80.3)	**0.029**
	Positive(5-9)	1 (1.7)		34 (72.3)		7 (15.6)	12 (36.4)	2 (7.1)	15 (19.7)	
Notch3 status	Negative (0-4)	58 (98.3)	81 (100.0)	24 (51.1)	**<0.001**	42 (91.3)	26 (78.8)	26 (92.9)	64 (85.3)	0.295
	Positive(5-9)	1 (1.7)		23 (48.9)		4 (8.7)	7 (21.2)	2 (7.1)	11 (14.7)	
Gli (cutoff at 3)	Negative	35 (59.3)	51 (64.6)	**40 (85.1)**	**0.012**	33 (71.7)	21 (63.6)	2 (7.1)	71 (93.4)	**<0.001**
	Positive	24 (40.7)	28 (35.4)	7 (14.9)		13 (28.3)	12 (36.4)	26 (92.9)	5 (6.6)	
Patched-1 (cutoff at 3)	Negative	36 (61.0)	61 (78.2)	26 (55.3)	**0.016**	30 (65.2)	1 (3.0)	25 (89.3)	67 (88.2)	**<0.001**
	Positive	23 (39.0)	17 (21.8)	**21 (44.7)**		16 (34.8)	32 (97.0)	3 (10.7)	9 (11.8)	
Shh (cutoff at 3)	Negative	2 (3.4)	3 (3.9)	0 (0)	0.405			1 (3.6)	4 (5.3)	0.243
	Positive	57 (96.6)	74 (96.1)	47 (100.0)		46 (100.0)	33 (100.0)	27 (96.4)	71 (94.7)	
Smo (cutoff at 3)	Negative	36 (63.2)	43 (56.6)	31 (66.0)	0.543	1 (2.2)	19 (57.6)	27 (96.4)	65 (85.5)	**<0.001**
	Positive	21 (36.8)	33 (43.4)	16 (34.0)		45 (97.8)	14 (42.4)	1 (3.6)	11 (14.5)	
MMR final status	Deficiency	24 (44.4)	44 (57.9)	12 (27.9)	**0.007**	22 (48.9)	10 (30.3)	12 (50.0)	35 (50.0)	0.258
	Proficiency	30 (55.6)	32 (42.1)	**31 (72.1)**		23 (51.1)	23 (69.7)	12 (50.0)	35 (50.0)	
					Clinicopathological parameters					
Age (median cut-off)	≤64	35 (59.3)	32 (39.5)	27 (57.4)	**0.036**	20 (43.5)	18 (54.5)	14 (50.0)	38 (50.0)	0.8
	>64	24 (40.7)	49 (60.5)	20 (42.6)		26 (56.5)	15 (45.5)	14 (50.0)	38 (50.0)	
Type	1	54 (91.5)	73 (91.3)	**28 (59.6)**	**<0.001**	37 (80.4)	24 (72.7)	**28 (100.0)**	62 (82.7)	0.038
	2	5 (8.5)	7 (8.8)	19 (40.4)		9 (19.6)	9 (27.3)	0 (0)	13 (17.3)	
Stage (binary)	I-II	47 (82.5)	68 (88.3)	23 (48.9)	**<0.001**	38 (86.4)	23 (71.9)	23 (82.1)	49 (67.1)	0.094
	III-IV	10 (17.5)	9 (11.7)	**24 (51.1)**		6 (13.6)	9 (28.1)	5 (17.9)	24 (32.9)	
Grade	I	20 (34.5)	28 (35.0)	4 (8.9)	**<0.001**	11 (23.9)	8 (25.8)	11 (39.3)	20 (26.7)	0.063
	II	29 (50.0)	39 (48.8)	14 (31.1)		23 (50.0)	9 (29.0)	15 (53.6)	33 (44.0)	
	III	9 (15.5)	13 (16.3)	**27 (60.0)**		12 (26.1)	14 (45.2)	2 (7.1)	22 (29.3)	
Depth of invasion	<50%	26 (45.6)	43 (56.6)	21 (44.7)	0.319	24 (55.8)	16 (50.0)	13 (46.4)	35 (47.9)	0.838
	>50%	31 (54.4)	33 (43.4)	26 (55.3)		19 (44.2)	16 (50.0)	15 (53.6)	38 (52.1)	
