# Supplementary material for: Mismatch repair deficiency and aberrations in the Notch and Hedgehog pathways are of prognostic value in patients with endometrial cancer
Source: PLoS One. 2018 Dec 6;13(12):e0208221. doi: 10.1371/journal.pone.0208221 (PMC6283658; doi:10.1371/journal.pone.0208221)

S1 Fig: Representative examples of positive and negative classical IHC markers in endometrial carcinomas.

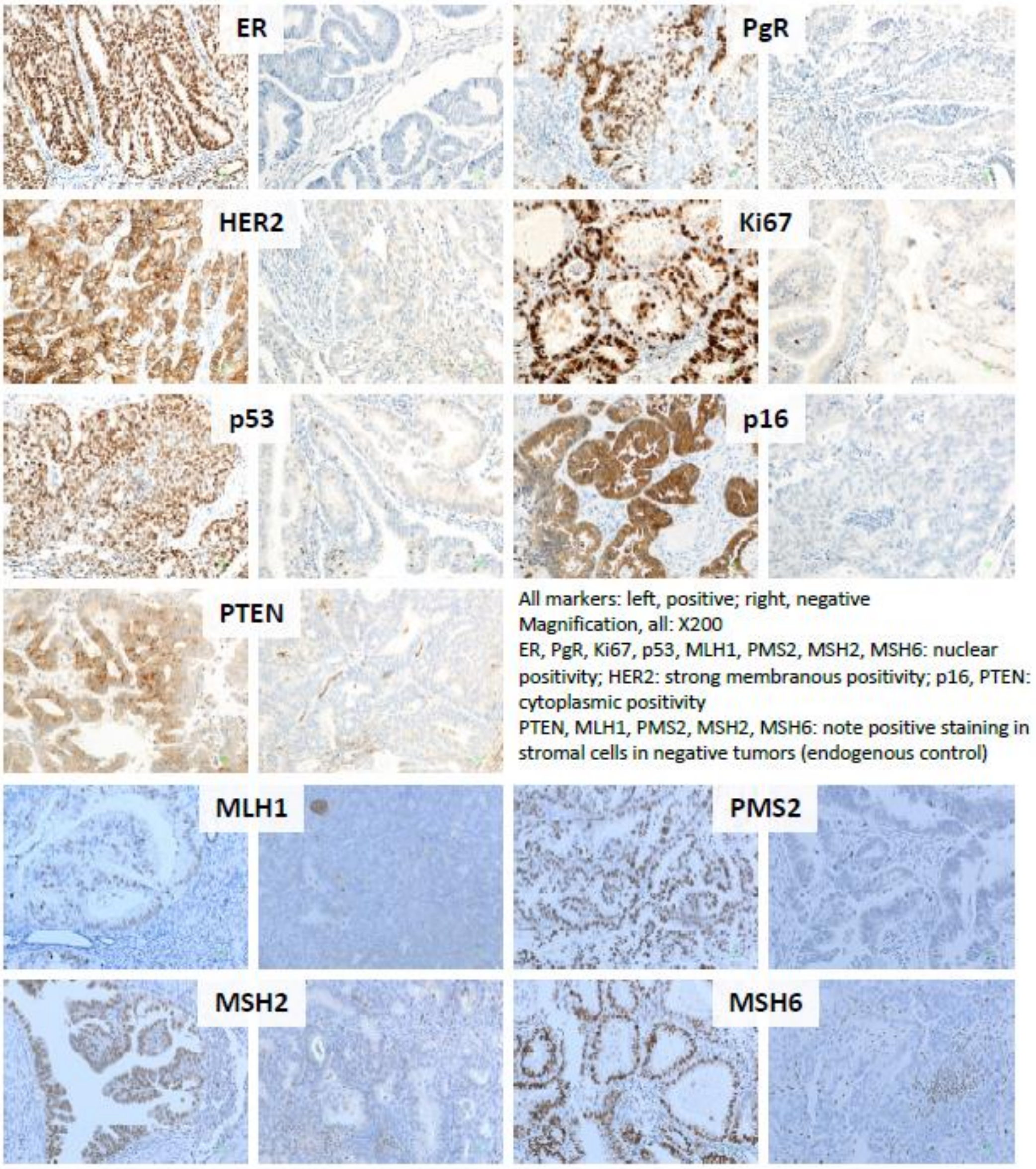

Supplement: S1 Fig — (PDF) [file pone.0208221.s009.pdf]
